# Linalool in cancer pharmacology: pharmacokinetic limitations and preclinical cytotoxic evidence for translational development

**DOI:** 10.3389/fphar.2026.1891927

**Published:** 2026-08-04

**Authors:** Asad Abbas, Muhammad Bilal, Fatima Jafar, Ralf Weiskirchen, Adnan Amjad, Muhammad Khurram Afzal, Sawaira Bibi, Anza Saleem, Muhammad Israr, Aimen Mazhar, Bipindra Pandey

**Affiliations:** 1 Department of Human Nutrition, Faculty of Food Science and Nutrition, Bahauddin Zakariya University, Multan, Pakistan; 2 Multan College of Nutritional Sciences, Multan Medical and Dental College, Multan, Pakistan; 3 Faculty of Food Science and Nutrition, Bahauddin Zakariya University, Multan, Pakistan; 4 Institute of Molecular Pathobiochemistry, Experimental Gene Therapy and Clinical Chemistry (IFMPEGKC), RWTH University Hospital Aachen, Aachen, Germany; 5 Faculty of Allied Health Sciences, University of Southern Punjab, Multan, Pakistan; 6 Department of Pharmacy, Madan Bhandari Academy of Health Sciences, Hetauda, Bagmati, Nepal

**Keywords:** apoptosis, bioavailability, cancer cell lines, cytotoxicity, evidence appraisal, extraction methods, linalool, pharmacokinetics

## Abstract

Cancer pharmacology increasingly evaluates plant-derived metabolites as lead compounds, but preclinical cytotoxicity should not be equated with clinical anticancer efficacy. Linalool, an acyclic monoterpene alcohol present in several aromatic plants and essential oils, has been investigated as an isolated compound and as a component of linalool-containing preparations. This critical narrative review evaluates pharmacokinetic evidence related to linalool, delivery considerations, the relevance of extraction methods, and preclinical cancer-model findings, with explicit separation of computational predictions, *in vitro* assays, animal studies, and human pharmacokinetic and safety evidence. Available primary evidence mainly supports cytotoxic, antiproliferative, pro-apoptotic, anti-inflammatory, and redox-modulating effects in cancer cell models, with limited evidence from animal studies and no established clinical anticancer efficacy. Reported endpoints include reactive oxygen species modulation, mitochondrial dysfunction, caspase activation, cell-cycle arrest, and changes in MAPK/ERK, PI3K/AKT, Ras/AKT/mTOR, JAK/STAT, NF-κB, and Bax/Bcl-2-related markers. However, many *in vitro* studies use high micromolar or millimolar concentrations that may exceed systemic exposures achievable after oral administration. Translational development therefore requires chemically standardized preparations, molar dose reporting, exposure–response validation, appropriate positive and negative controls, reproducible animal studies, and clinical studies focused initially on safety, pharmacokinetics, and realistic therapeutic endpoints.

## 1 Introduction

Cancer remains a complex and multifactorial disease characterized by uncontrolled cellular proliferation, invasion, and metastasis, resulting from genetic mutations, epigenetic alterations, and the dysregulation of processes involved in cell division, apoptosis, DNA repair, and immune regulation (Hanahan, 2022). Despite substantial advances in prevention, diagnosis, and treatment, cancer continues to represent a major global health burden. The Global Cancer Observatory estimated approximately 19.3 million new cancer cases and nearly 10 million cancer-related deaths worldwide in 2020 (International Agency for Research on Cancer, 2026), highlighting the continuing need for improved therapeutic strategies and better-tolerated treatment approaches (Sung et al., 2021). Natural products have historically contributed significantly to oncology drug discovery, with clinically successful agents such as paclitaxel from *Taxus brevifolia*, camptothecin-derived topoisomerase I inhibitors from *Camptotheca acuminata*, Vinca alkaloids from *Catharanthus roseus*, and podophyllotoxin-derived compounds demonstrating the value of plant-derived molecules as pharmacological leads (Wani et al., 1971; Wall et al., 1966; Noble et al., 1958; Cutts et al., 1960). However, the success of these compounds does not establish therapeutic potential for other natural metabolites without evidence demonstrating appropriate exposure, validated mechanisms, biological selectivity, and clinical relevance. Therefore, anticancer claims based on natural products require careful interpretation using pharmacologically meaningful concentrations, appropriate experimental controls, chemical characterization, and evidence-based translational assessment (Olaku and White, 2011; Api et al., 2015).

Linalool, an acyclic monoterpene alcohol (3,7-dimethyl-1,6-octadien-3-ol), has attracted increasing interest because of its diverse biological activities, including antimicrobial, anti-inflammatory, antioxidant, and cancer cell–cytotoxic effects. It is naturally present in more than 200 plant species, including members of the *Cinnamomum* (Lauraceae), *Coriandrum* (Apiaceae), *Lavandula* (Lamiaceae), and *Citrus* (Rutaceae) genera, and is commonly identified in essential oils, plant extracts, and floral volatile compounds (Jiang et al., 2015; Pereira et al., 2018a). Experimental studies have reported that linalool may influence cancer-related processes, including apoptosis induction, oxidative stress regulation, cell-cycle arrest, and modulation of signaling pathways associated with tumor progression (Chang et al., 2015; Ni et al., 2021). However, linalool is investigated in different forms, including purified compounds, linalool-rich essential oils, botanical extracts, and delivery-based formulations, and these sources cannot be considered equivalent. Biological effects observed with essential oils or extracts should not be attributed exclusively to linalool unless the chemical composition, botanical identity, and linalool concentration are adequately characterized. This distinction is essential for accurately evaluating the specific pharmacological contribution of linalool.

Linalool is chiral and occurs mainly as two enantiomers, R-(−)-linalool, also known as licareol, and S-(+)-linalool, also known as coriandrol. The relative abundance of these stereoisomers varies among botanical sources and may influence odor characteristics, receptor interactions, metabolism, and biological responses (Cheng et al., 2015). Variations in enantiomeric composition among plant sources can influence aroma characteristics, receptor interactions, metabolism, and potentially pharmacological responses. However, many pharmacological studies evaluating anticancer-related effects do not report stereochemical composition, creating uncertainty regarding whether observed biological activities are attributable to a specific enantiomer or the racemic mixture.

Although previous reviews have summarized the broad biological activities of linalool, including its antimicrobial, antioxidant, anti-inflammatory, and anticancer-related properties, many discussions have not sufficiently addressed whether reported cytotoxic effects are pharmacokinetically achievable or clinically interpretable. The relevance of preclinical anticancer findings depends not only on biological activity but also on exposure feasibility, bioavailability, metabolism, formulation strategy, and the relationship between experimentally effective concentrations and achievable tissue or plasma levels. Linalool exhibits pharmacokinetic limitations associated with lipophilicity, rapid metabolism, and restricted systemic availability following oral administration, creating an important translational gap between *in vitro* observations and potential therapeutic application (Al-Harrasi et al., 2022; Wang et al., 2023). Although advanced delivery approaches, including nanoencapsulation, lipid carriers, and inclusion complexes, have been explored to improve stability and bioavailability, their ability to achieve therapeutically relevant anticancer exposure requires further validation (Shi et al., 2016; Pereira et al., 2018b). Therefore, cytotoxic effects observed at high micromolar or millimolar concentrations should be interpreted cautiously unless supported by exposure-response relationships, appropriate *in vivo* models, and pharmacokinetic evidence.

The present narrative review aims to provide a comprehensive evaluation of linalool-related anticancer evidence by integrating pharmacokinetic characteristics, delivery considerations, extraction methods, and preclinical cancer-model findings. Unlike previous broad reviews, this study specifically distinguishes evidence derived from pure linalool from that derived from linalool-containing preparations, separates computational predictions from experimentally validated mechanisms, and evaluates the translational plausibility of reported anticancer effects. Particular emphasis is placed on determining whether observed cytotoxic, antiproliferative, pro-apoptotic, anti-inflammatory, and signaling-related effects can be reconciled with achievable biological exposure. By critically assessing evidence from breast, ovarian, lung, hepatocellular carcinoma, leukemia, cervical, and other cancer models, this review does not present linalool as an established anticancer agent but instead evaluates its potential as a preclinical lead or adjunct candidate requiring further pharmacological validation. Particular emphasis is placed on the gap between concentrations used *in vitro* and systemic exposures reported in pharmacokinetic studies. Therefore, this review does not present linalool as an established anticancer agent, but rather evaluates the strength, limitations, and translational relevance of the current preclinical evidence.

## 2 Search methodology and evidence appraisal

This critical narrative review was based on structured literature identification and evidence appraisal. Searches were conducted in PubMed/MEDLINE, Scopus, Web of Science, ScienceDirect, and Google Scholar from database inception to February 2026. The core search strategy combined linalool-related terms with cancer, pharmacology, and extraction terms. The PubMed search string was (“linalool” OR “3,7-dimethyl-1,6-octadien-3-ol” OR “monoterpene alcohol”) AND (cancer OR carcinoma OR tumor OR tumour OR neoplasm OR leukemia OR glioblastoma OR breast OR ovarian OR lung OR hepatocellular OR cervical OR colon OR prostate OR oral) AND (cytotoxicity OR apoptosis OR “cell cycle” OR proliferation OR pharmacokinetic OR bioavailability OR “drug delivery” OR extraction). For Scopus and Web of Science, the same terms were searched in the title, abstract, and keywords using Boolean operators. A second search string was used for extraction studies (linalool OR monoterpene) AND (“steam distillation” OR hydrodistillation OR “supercritical fluid extraction” OR “microwave-assisted extraction” OR “ultrasound-assisted extraction” OR Soxhlet OR “subcritical water extraction” OR “enzyme-assisted extraction”).

Inclusion criteria were: original *in vitro*, *in vivo*, pharmacokinetic, pharmacological, analytical, or formulation studies involving linalool as a pure metabolite or a chemically characterized linalool-containing preparation; studies reporting cancer-cell cytotoxicity, proliferation, apoptosis, cell-cycle, inflammatory, pharmacokinetic, bioavailability, or extraction outcomes; and clinical or human studies reporting pharmacokinetic, safety, tolerability, or exposure data relevant to translational assessment. Computational studies were included only for contextual discussion and were coded as hypothesis-generating unless accompanied by experimental validation.

Exclusion criteria were: non-English publications, inaccessible full texts, homeopathic preparations, studies in which linalool was not separable from other metabolites or mixtures, studies reporting only general antioxidant chemical assays without pharmacological relevance, duplicate records, and papers that did not report sufficient methodological information to identify the model or material tested. Reviews were used for background only and were not treated as primary pharmacological evidence.

For interpretation, studies were grouped into four material categories: free linalool, linalool-rich essential oils, botanical extracts, and linalool-containing delivery systems or nanoformulations. Effects observed with essential oils or extracts were attributed to the whole preparation unless quantified linalool content and dose equivalence were reported. Effects observed with nanoparticles, liposomes, lipid carriers, peptide-conjugated systems, or other delivery platforms were attributed to the formulation system unless the study directly compared free linalool with the carrier and the loaded formulation. A total of 517 studies were initially identified. After duplicate removal and title/abstract screening, 381 records were selected for full-text assessment. 257 records were excluded during the full-text review, and 124 articles were included in the final synthesis. Records were screened to identify primary studies relevant to linalool pharmacokinetics, delivery, extraction, and cancer-related experimental models. Because this was a critical narrative review rather than a systematic review, the selection process is described narratively.

To strengthen methodological transparency, a structured quality and risk-of-bias appraisal was performed for the included linalool cancer-model studies. Each study was assessed for material category, material identity, reported purity, dose/concentration, exposure duration, positive control, negative or vehicle control, selectivity against non-cancer cells, formulation details, statistical robustness, and overall confidence. Studies reporting clear material characterization, appropriate controls, dose–response testing, non-cancer selectivity, and adequate statistical reporting were assigned higher confidence, whereas studies with incomplete reporting or limited experimental controls were interpreted cautiously. The results of this appraisal are presented in Supplementary Table S1, which provides a structured quality and risk-of-bias appraisal of included linalool cancer-model studies.

For the selection of appropriate articles, we assessed whether the original study reported the full botanical name, family, plant part, extraction method, chemical profile, and quantified linalool content. When such information was absent, this was treated as a limitation rather than as evidence supporting a specific linalool-mediated effect.

## 3 Linalool pharmacokinetics, bioavailability, and delivery considerations

Linalool has received pharmacological attention because of reported anxiolytic, anti-inflammatory, antimicrobial, and cytotoxic activities in experimental systems. Its widespread use in food, fragrance, and cosmetic products does not, by itself, establish therapeutic value, and any development as a medicinal lead requires assessment of exposure, bioavailability, safety, metabolism, and potential drug interactions. Oral linalool generally shows moderate to low systemic availability (Goswami et al., 2025; Wang et al., 2023). This profile is attributed to its lipophilicity, tissue distribution, and hepatic metabolism, including cytochrome P450-mediated first-pass metabolism (Al-Harrasi et al., 2022). Inhalation and dermal absorption have also been reported, with inhalation sometimes producing faster central nervous system effects (Brain et al., 2022).

For drug development purposes, nanoencapsulation, complexation, and chemical modification have been explored to improve stability and bioavailability. An oral lavender essential oil formulation containing 36.8% linalool and 34.2% linalyl acetate has been administered in human clinical studies for anxiolytic outcomes rather than anticancer efficacy (Yap et al., 2019). Experimental formulations of linalool have been evaluated as adjunctive systems in preclinical models and have shown low toxicity with anti-inflammatory or cytotoxic endpoints in selected disease models (An et al., 2021). After oral administration, linalool reached a mean maximum concentration (C_max_) of 85.5 ± 42.6 ng/mL at approximately 1 hour (T_max_), with oral clearance (CL/F) of 385 ± 287 L/h, a volume of distribution (Vd/F) of 1495 ± 898.4 L, a half-life of 3.9 ± 2.9 h, detectable plasma concentrations up to 8 hours, and serum AUC values of 442 ± 243 h·ng/mL (Wang et al., 2023).

To assess translational plausibility, reported *in vitro* active concentrations were compared with available human pharmacokinetic exposure. Using a molecular weight of 154.25 g/mol, a plasma C_max_ of 85.5 ng/mL corresponds to approximately 0.55 µM. This value is substantially below many reported cytotoxic concentrations, including 100–400 µM in T-47D breast cancer cells and 2.0 mM in A549 lung cancer cells. Therefore, *in vitro* effects observed at high micromolar or millimolar concentrations should be interpreted cautiously unless supported by formulation-enhanced exposure, local delivery, tissue accumulation, or *in vivo* exposure-response data (Wang et al., 2023). To further evaluate translational plausibility, reported anticancer concentrations of linalool were compared with human systemic exposure following oral administration. As summarized in Table 1, many *in vitro* studies employ concentrations substantially higher than the plasma C_max_ achieved in humans. Although some models report activity at lower micromolar concentrations, these findings require confirmation under physiologically relevant conditions, considering factors such as protein binding, metabolism, tissue distribution, and exposure duration. Furthermore, nanoparticle-based studies should be interpreted separately because enhanced cellular uptake may result from formulation characteristics rather than from linalool itself.

**TABLE 1:** Comparison of reported experimental anticancer concentrations of linalool with human plasma exposure after oral administration.

Cancer experimental model	Linalool concentration	Reported human plasma exposure after oral administration	Translational interpretation	References
T-47D breast cancer cells	100–400 µM	Human plasma C_max_ ≈ 0.55 µM after 100 mg oral linalool administration	The experimental concentrations are approximately 180–720-fold higher than reported human systemic exposure, suggesting limited direct translational feasibility without improved delivery strategies or tissue-specific accumulation	Hsu et al. (2014); Wang et al. (2023)
A549 lung cancer cells	2.0 mM	Human plasma C_max_ ≈ 0.55 µM	Millimolar concentrations exceed achievable systemic exposure following conventional oral dosing and require cautious interpretation regarding clinical relevance	Rodenak-Kladniew et al. (2020); Wang et al. (2023)
U937 leukemia cells	IC_50_ ≈ 2.59 µM	Human plasma C_max_ ≈ 0.55 µM	The effective concentration is closer to reported plasma exposure compared with other models; however, confirmation under physiological conditions considering protein binding, metabolism, and exposure duration is required	Chang et al. (2015); Wang et al. (2023)
HeLa cervical cancer cells	IC_50_ ≈ 11.02 µM	Human plasma C_max_ ≈ 0.55 µM	Although lower than many reported cytotoxic concentrations, the experimental concentration remains substantially higher than measured systemic exposure	Chang et al. (2015); Wang et al. (2023)
OECM-1 oral cancer cells	IC_50_ ≈ 10 µM	Human plasma C_max_ ≈ 0.55 µM	The concentration exceeds human plasma exposure by approximately 18-fold; pharmacokinetic–pharmacodynamic validation is required	Pan and Zhang (2019); Wang et al. (2023)
HepG2 hepatocellular carcinoma cells	2 µM	Human plasma C_max_ ≈ 0.55 µM	The concentration is within a closer pharmacological range but requires validation considering tissue distribution, metabolism, and selective toxicity	Usta et al. (2009); Wang et al. (2023)
MCF-7 breast cancer cells with linalool-loaded gold nanoparticles	10 μg/mL LIN-GNPs and LIN-GNPs-CALNN	Human plasma C_max_ ≈ 0.55 µM (free linalool)	Observed activity may reflect nanoparticle-mediated uptake or carrier effects rather than linalool alone; direct comparison with free linalool exposure is not possible	Jabir et al. (2019); Wang et al. (2023)
SF-767 glioblastoma cells	33.14 and 22.12 μg/mL (linalool and linalool-based nanoconjugates)	Human plasma C_max_ ≈ 0.55 µM	Nanoconjugate-associated effects require separation of linalool-specific activity from formulation-dependent enhancement	Manzoor et al. (2025); Wang et al. (2023)

Abbreviations used: A549, human lung adenocarcinoma epithelial cell line; CALNN, cysteine-alanine-leucine-asparagine-asparagine peptide; C_max_, maximum plasma concentration; HeLa, human cervical cancer cell line; HepG2, human hepatocellular carcinoma cell line; IC_50_, half-maximal inhibitory concentration; LIN-GNPs, linalool-loaded gold nanoparticles; LIN-GNPs-CALNN, CALNN-functionalized linalool-loaded gold nanoparticles; MCF-7, Michigan Cancer Foundation-7 breast cancer cell line; OECM-1, human oral squamous carcinoma cell line; SF-767, human glioblastoma cell line; T-47D, human breast cancer cell line; U937, human leukemia/monocytic lymphoma cell line.

Linalool exposure following linalyl acetate administration represented approximately one-tenth of that following linalool administration (64.9 ± 78.0 vs. 442 ± 243 h·ng/mL). According to the aforementioned study (Nöldner et al., 2011), an estimated linalool dose of approximately 75 mg can be inferred to 28.9 mg/kg body weight, assuming an average body weight of 0.4 kg for rats. Thus, linalool oral bioavailability appears slightly higher in humans (100 mg dose, 85 ng/mL C_max_) compared with rats (75 mg dose, 33 ng/mL C_max_) (Wang et al., 2023). The preparation of linalool-loaded nanostructured lipid carriers or β-cyclodextrin complexes significantly improved its oral bioavailability in rats (Shi et al., 2016). Linalool complexed with β-cyclodextrin significantly enhanced systemic bioavailability, increasing it approximately twenty-fold and extending the circulation time. In acute experiments, intravenous administration of linalool and its complexes (50 mg/kg) decreased blood pressure–induced arterial alterations, exhibiting antihypertensive and cardioprotective activities (Camargo et al., 2025). Experimental studies have also demonstrated that linalool can relax contractions induced by prostaglandin F2 (PGF2) in isolated rat aortic rings and cause endothelium-dependent vasodilation in mice. These effects are thought to be mediated through activation of soluble guanylate cyclase (sGC) and potassium channels, along with inhibition of calcium influx (Kang and Seol, 2015).

In the case of linalool, five pharmacophoric features have been identified: two hydrophobic groups in the terpene side chain, a dual region (acting as either a hydrogen bond acceptor or donor) in one terminal zone of the molecule, and two atoms in the other terminal zone (Soares-Santos et al., 2025). This review also described the pharmacokinetic characteristics of linalool and its potential relevance in drug development (Singh and Meena, 2022). Nanotechnology has been used to enhance the properties of linalool, including by reducing volatility and improving bioavailability. This has been achieved through the use of metallic nanoparticles (gold, zinc, and their oxides) (Alkhaibari et al., 2024; Jabir et al., 2018), polymeric nanoparticles (polycaprolactone and polyhydroxyethyl methacrylate) (Devin et al., 2025), liposomes (Wi et al., 2020), solid lipid nanoparticles (Pereira et al., 2018b), nanostructured lipid carriers (Shi et al., 2016), nanoemulsions (Zhong et al., 2021), emulgels (Akram et al., 2021), organogels (Yogev and Mizrahi, 2020), and nanofibers (Celebioglu et al., 2025).

Linalool may interact with other medications because of its potential effects on metabolic enzymes and the central nervous system. It may alter cytochrome P450 enzyme activity and thereby influence the metabolism of co-administered drugs, including sedatives, antidepressants, and antiepileptic agents. Because linalool has sedative and anxiolytic properties, additive effects with central nervous system depressants such as benzodiazepines and alcohol should be considered (Weston-Green et al., 2021). In cancer-related research, linalool should therefore be regarded as a preclinical lead or adjunctive candidate requiring exposure-response validation rather than as an established cancer-related drug. Novel delivery methods may improve stability, targeting, and systemic exposure; however, therapeutic relevance depends on whether achievable concentrations correspond to those required for cytotoxic or pro-apoptotic effects in experimental models (Soares-Santos et al., 2025).

In the human pharmacokinetic study, systemic exposure to linalool after administration of linalyl acetate was lower than that observed after direct linalool administration, as reflected by the reported AUC values. However, a direct comparison between rat and human oral bioavailability is not appropriate because the available studies differ in species, dose, formulation, route/exposure conditions, and pharmacokinetic design. Therefore, the rat data should be interpreted only as preclinical formulation-related evidence, not as evidence that linalool has higher oral bioavailability in humans (Wang et al., 2023).

The values below were retained because they are either reported directly in the manuscript or verified from accessible source records. Wang et al. (2023) report the human single-dose linalool PK values, including C_max_ of 85.49 ± 42.56 ng/mL, T_max_ of 1.15 ± 0.24 h, a half-life of 3.93 ± 2.94 h, AUC of 441.86 ± 242.69 h·ng/mL, and CL/F of 384.83 ± 287.04 L/h. Shi et al. (2016) report that linalool-loaded nanostructured lipid carriers increased C_max_ from 1915.45 to 2182.45 ng/mL, AUC0–t from 76,003.40 to 298,948.46 ng·min/mL, and relative bioavailability to 393.34%. Camargo et al. (2025) provide NCA parameters for free linalool and linalool/β-cyclodextrin in rats, including AUC, half-life, clearance, absolute bioavailability, and relative bioavailability, but not C_max_ or T_max_ (Table 2). The following cancer-model synthesis should therefore be read in light of pharmacokinetic plausibility. Particular attention is given to whether experimentally active concentrations are achievable through systemic exposure, local delivery, or formulation-based enhancement.

**TABLE 2:** Pharmacokinetic parameters of linalool and linalool-containing formulations.

Study	Model	Formulation	Route	Dose	C_max_	T_max_	AUC	Half-life	Clearance	Bioavailability	Key limitation	References
Human single-dose linalool PK	Healthy human volunteers	Linalool in hypromellose capsules with olive oil	Oral	100 mg total linalool	85.49 ± 42.56 ng/mL	1.15 ± 0.24 h	441.86 ± 242.69 h · ng/mL	3.93 ± 2.94 h	CL/F: 384.83 ± 287.04 L/h	ND; no IV comparator	Healthy-volunteer PK only; not an anticancer efficacy study	Wang et al. (2023)
Human linalyl acetate arm	Healthy human volunteers	Linalyl acetate in hypromellose capsules with olive oil; measured as linalool exposure	Oral	90 mg total linalyl acetate	NR; linalool levels mostly below LLOQ	NR	64.9 ± 78.0 h · ng/mL for linalool exposure	NR	NR	ND; linalool exposure approximately one-tenth of direct linalool dosing	Indirect precursor exposure; many samples below quantification limit	Wang et al. (2023)
Rat single-dose comparison	Rats	Pure linalool	Oral	28.9 mg/kg	33 ng/mL	NR	NR	NR	NR	Lower exposure than lavender essential oil formulation	Conference abstract; limited accessible PK detail	Nöldner et al. (2011)
Rat single-dose comparison	Rats	Linalyl acetate	Oral	36.8 mg/kg	10 ng/mL, measured as linalool	NR	NR	NR	NR	Lower linalool exposure than direct linalool and lavender oil	Conference abstract; limited accessible PK detail	Nöldner et al. (2011)
Rat single-dose comparison	Rats	Lavender essential oil/Silexan	Oral	100 mg/kg	77 ng/mL, measured as linalool	NR	NR	NR	NR	Reported higher relative oral exposure than pure linalool or linalyl acetate	Conference abstract; complete NCA parameters not available	Nöldner et al. (2011)
Nanostructured lipid carrier comparison	Rats	Free linalool solution	Oral gavage	300 mg/kg	1915.45 ng/mL	NR	76,003.40 ng · min/mL	NR	NR	Reference comparator, 100%	High animal dose; exact Tmax/half-life/clearance should be verified from full text	Shi et al. (2016)
Nanostructured lipid carrier comparison	Rats	Linalool-loaded nanostructured lipid carriers	Oral gavage	300 mg/kg	2182.45 ng/mL	Reported longer than free linalool; exact value NR	298,948.46 ng · min/mL	Reported longer than free linalool; exact value NR	NR	Relative bioavailability: 393.34%	Formulation PK in rats only; not cancer-targeted PK	Shi et al. (2016)
β-Cyclodextrin comparison	Wistar rats	Free linalool	Intravenous	50 mg/kg	NR	NA	25.28 ± 18.65 μg · h/mL	6.60 ± 4.04 h	3.03 ± 1.67 L/h/kg	NA for IV	Cardiovascular model; not a cancer model	Camargo et al. (2025)
β-Cyclodextrin comparison	Wistar rats	Free linalool	Oral	100 mg/kg	NR	NR	0.04 ± 0.02 μg · h/mL	8.64 ± 4.44 h	5.06 ± 2.27 L/h/kg	Fabs: 0.003% ± 0.001%	Extremely low oral exposure; C_max_/T_max_ not reported	Camargo et al. (2025)
β-Cyclodextrin comparison	Wistar rats	Linalool/β-cyclodextrin inclusion complex	Oral	100 mg/kg	NR	NR	90.34 ± 34.36 μg · h/mL	12.08 ± 4.27 h	1.27 ± 0.55 L/h/kg	Fabs: 1.26% ± 0.48%; Frel: 19.53 ± 7.42	Cardiovascular formulation study; no anticancer exposure-response validation	Camargo et al. (2025)
β-Cyclodextrin comparison	Spontaneously hypertensive rats	Linalool/β-cyclodextrin inclusion complex	Oral	50 mg/kg	NR	NR	51.28 ± 19.29 μg · h/mL	8.95 ± 3.52 h	1.08 ± 0.62 L/h/kg	Fabs: 1.78% ± 0.37%; Frel: 17.84 ± 6.75	Disease model was hypertension, not cancer	Camargo et al. (2025)

Abbreviations used: NR, not reported; ND, not determined; NA, not applicable; NCA, noncompartmental analysis; LLOQ, lower limit of quantification; PK, pharmacokinetics; CL/F = apparent oral clearance; Fabs = absolute bioavailability; Frel = relative bioavailability. AUC, units are retained as reported in each study. For Shi et al. (2016), AUC, reported in ng·min/mL can be converted to h·ng/mL by dividing by 60.

## 4 Preclinical cytotoxic and pro-apoptotic evidence across cancer models

Most cancer-related studies of linalool are early-stage *in vitro* assays, supported by fewer animal studies. Therefore, this section summarizes cytotoxic, antiproliferative, pro-apoptotic, anti-inflammatory, and antimigratory endpoints according to model type, dose, exposure duration, and evidence tier. Table 3 summarizes reported endpoints and their evidence tiers across cancer models. Importantly, the available evidence remains largely preclinical. *In vitro* reductions in viability, ROS modulation, caspase activation, cell-cycle arrest, or apoptosis-associated morphology should not be interpreted as evidence of therapeutic anticancer efficacy unless supported by pharmacokinetically achievable exposure, selectivity against non-cancer cells, reproducible animal data, and well-designed clinical studies.

**TABLE 3:** Evidence hierarchy and qualified interpretation of reported linalool effects in cancer-related models.

Cancer/model context	Evidence tier or study type	Reported endpoint(s)	Qualified interpretation and Reference	References
Lung cancer	Computational docking/ADMET	Predicted cell-cycle, apoptosis, autophagy, and ADMET associations	Hypothesis-generating only; not mechanistic proof without bench validation	Singh and Meena (2022)
Lung inflammation/lung cancer context	*In vivo* cigarette-smoke-induced model	Reduced TNF-α, IL-6, IL-1β, IL-8, MCP-1, and NF-κB activation	Preclinical anti-inflammatory evidence; not evidence of direct anticancer efficacy	Ma et al. (2015)
Hepatocellular carcinoma/HepG2	*In vitro* cell model	Reduced HepG2 viability; inhibited mitochondrial complexes I and II; increased ROS and reduced ATP and GSH	Cytotoxic and mitochondrial stress evidence *in vitro*	Usta et al. (2009)
Breast cancer	Computational/omics analysis with experimental context	Predicted apoptosis and G_0_/G_1_ cell-cycle associations; reduced viability reported in cell models	Docking/omics hypotheses should be separated from bench evidence	Zhang and Chen (2024)
Leukemia	*In vitro* cell model	Apoptosis-associated markers, p53/CDKI modulation, and G_0_/G_1_ arrest	Cytotoxic/pro-apoptotic *in vitro* evidence	Gu et al. (2010)
Ovarian cancer	*In vitro*/*in vivo* studies	ROS-associated apoptosis, caspase activation, and mitochondrial damage	Preclinical evidence; material identity and dose details must be assessed	Han et al. (2016)
Cervical cancer	*In vitro*	Cell-cycle arrest, apoptosis markers, and CDKI expression	Cytotoxic/pro-apoptotic *in vitro* evidence	Chang et al. (2015)
Prostate cancer	*In vitro*/*in vivo*	Reduced proliferation; apoptosis-associated and cell-cycle endpoints	Preclinical evidence, not clinical efficacy	Zhao et al. (2020)
Colon cancer	*In vitro*/*in vivo* context	Oxidative stress and hydroxyl radical production	Mechanistic relevance requires dose/exposure appraisal	Iwasaki et al. (2016)
Skin/UVB model	*In vivo*/cell model context	Reduced UVB-induced hyperplasia, lipid peroxidation, and edema	Photoprotective/anti-inflammatory endpoints; not cancer-treatment evidence	Gunaseelan et al. (2016)
Glioblastoma	In silico/*in vitro*	Cytotoxicity and inhibition index values in SF-767 cells	In silico claims should be qualified; *in vitro* cytotoxicity should be interpreted separately	Manzoor et al. (2025)
Oral cancer	*In vivo*/*in vitro*, as reported	Cell-cycle arrest, loss of MMP, PI3K/AKT signaling suppression	Preclinical evidence; verify model, controls, and exposure conditions in the original study	Pan and Zhang (2019)

Abbreviations used: ADMET, absorption, distribution, metabolism, excretion and toxicity; AKT, protein kinase B; ATP, adenosine triphosphate; CDKI, cyclin-dependent kinase inhibitor; CDKIs, cyclin-dependent kinase inhibitors; G_0_/G_1_ phase, resting (G_0_) and first gap (G_1_) phases of the cell cycle; GSH, glutathione; IL-1β, interleukin-1, beta; IL-6, interleukin-6; IL-8, interleukin-8; MCP-1, monocyte chemoattractant protein-1; MMP, mitochondrial membrane potential; NF-κB, nuclear factor κ-light-chain-enhancer of activated B cells; PI3K, phosphoinositide 3-kinase; ROS, reactive oxygen species; TNF-α, tumor necrosis factor-α; UVB, ultraviolet B radiation.

### 4.1 Breast cancer

Breast cancer (BC) is the most common cancer in women, with approximately 2.3 million cases reported worldwide in 2020. By 2070, annual BC cases are projected to rise to 4.4 million, with an estimated 685,000 deaths (Soerjomataram and Bray, 2021). Current treatment includes surgery, chemotherapy, radiotherapy, targeted therapy, immunotherapy, and endocrine therapy (Mampre et al., 2022). Plant metabolites can support lead identification, but cell-line cytotoxicity cannot be equated with therapeutic anticancer activity without pharmacokinetic plausibility, selectivity, and *in vivo* validation. Linalool has been investigated for antibacterial, anti-inflammatory, antioxidant, and cancer cell cytotoxic effects (Peana et al., 2002; Gong et al., 2020). Computational approaches, including molecular docking, molecular dynamics, multi-omics, and machine learning, may help generate hypotheses, but they do not establish a mechanism unless validated experimentally (Sahu et al., 2023).

**FIGURE 1:**
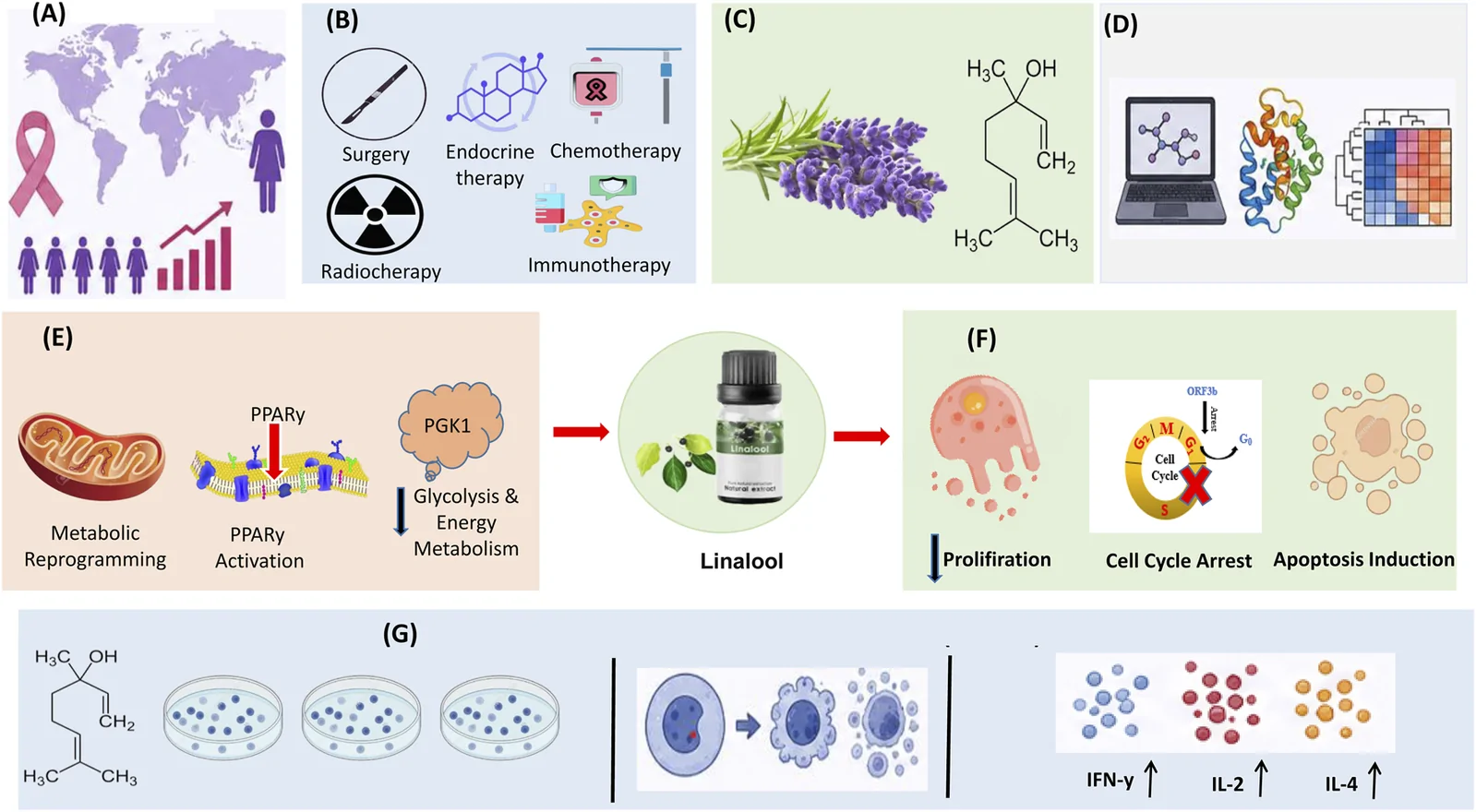
Linalool and its anticancer potential in breast cancer: From disease burden and therapeutic approaches to molecular mechanisms and experimental evidence. This figure shows **(A)** the global prevalence of breast cancer. **(B)** current treatment options for breast cancer. **(C)** the linalool structure. **(D)** computational approaches. **(E)** the proposed molecular mechanisms of linalool in breast cancer model, including linalool-mediated modulation of metabolic pathways, including peroxisome proliferator-activated receptor gamma (PPARγ) activation, metabolic reprogramming, and inhibition of glycolysis-related energy metabolism through regulation of phosphoglycerate kinase 1 (PGK1). **(F)** reported experimental outcomes, and **(G)** evidence from human breast cancer cell lines (*in vitro*).

In breast cancer models, linalool has been reported to affect metabolic and signaling pathways, including PPARγ activation and predicted interaction with phosphoglycerate kinase 1 (PGK1), which may influence glycolysis and energy metabolism. These findings should be interpreted as preclinical and, where based on computational or omics analyses, as hypothesis-generating. Reported experimental outcomes include reduced proliferation, migration, and invasion, together with cell-cycle arrest and apoptosis-related endpoints (Zhang and Chen, 2024). The proposed immunomodulatory mechanisms of linalool against BC are shown in Figure 1.

Studies in human breast cancer cell lines have reported that linalool reduces cell proliferation and induces apoptosis-associated morphological changes (Elbe et al., 2022). In that study, greater sensitivity was observed in MCF-7 cells, and Annexin V flow cytometry and microscopic analyses showed reduced viability in both MCF-7 and MDA-MB-231 cells. Apoptosis-associated morphological changes included cell shrinkage, membrane blebbing, and nuclear condensation. These endpoints support *in vitro* cytotoxic and pro-apoptotic activity, but they do not establish clinical anticancer efficacy. Treatment of T-47D breast cancer cells with 100–400 µM linalool for 24 h produced a dose-dependent reduction in viability, with an IC_50_ of approximately 224 µM. Flow cytometry further indicated apoptosis induction, G1-phase arrest, and an increased sub-G1 population. Increased IFN-γ, IL-2, IL-4, and TNF-α suggested immunomodulatory signaling, although the translational relevance of these concentrations requires further validation (Hsu et al., 2014). Linalool exhibits promising preclinical activity in breast cancer models through the suppression of proliferation, the induction of apoptosis, immune cytokine modulation, and the alteration of metabolic pathways including PPARγ-associated signaling and glycolysis. Strengths include the integration of cellular assays, flow cytometry, morphological evaluation, and computational approaches to generate mechanistic insights (Hsu et al., 2014; Elbe et al., 2022). However, the evidence remains limited by a reliance on *in vitro* systems, incomplete validation of predicted targets, and the use of concentrations exceeding reported systemic exposure levels (Zhang and Chen, 2024; Wang et al., 2023). Nanoparticle studies also require differentiation between linalool-specific and carrier-related effects (Jabir et al., 2019). Further pharmacokinetic–pharmacodynamic studies and selectivity assessments are required before considering linalool as a formulation-enhanced adjunct candidate (Wang et al., 2023; Pereira et al., 2018b).

### 4.2 Ovarian cancer

**FIGURE 2:**
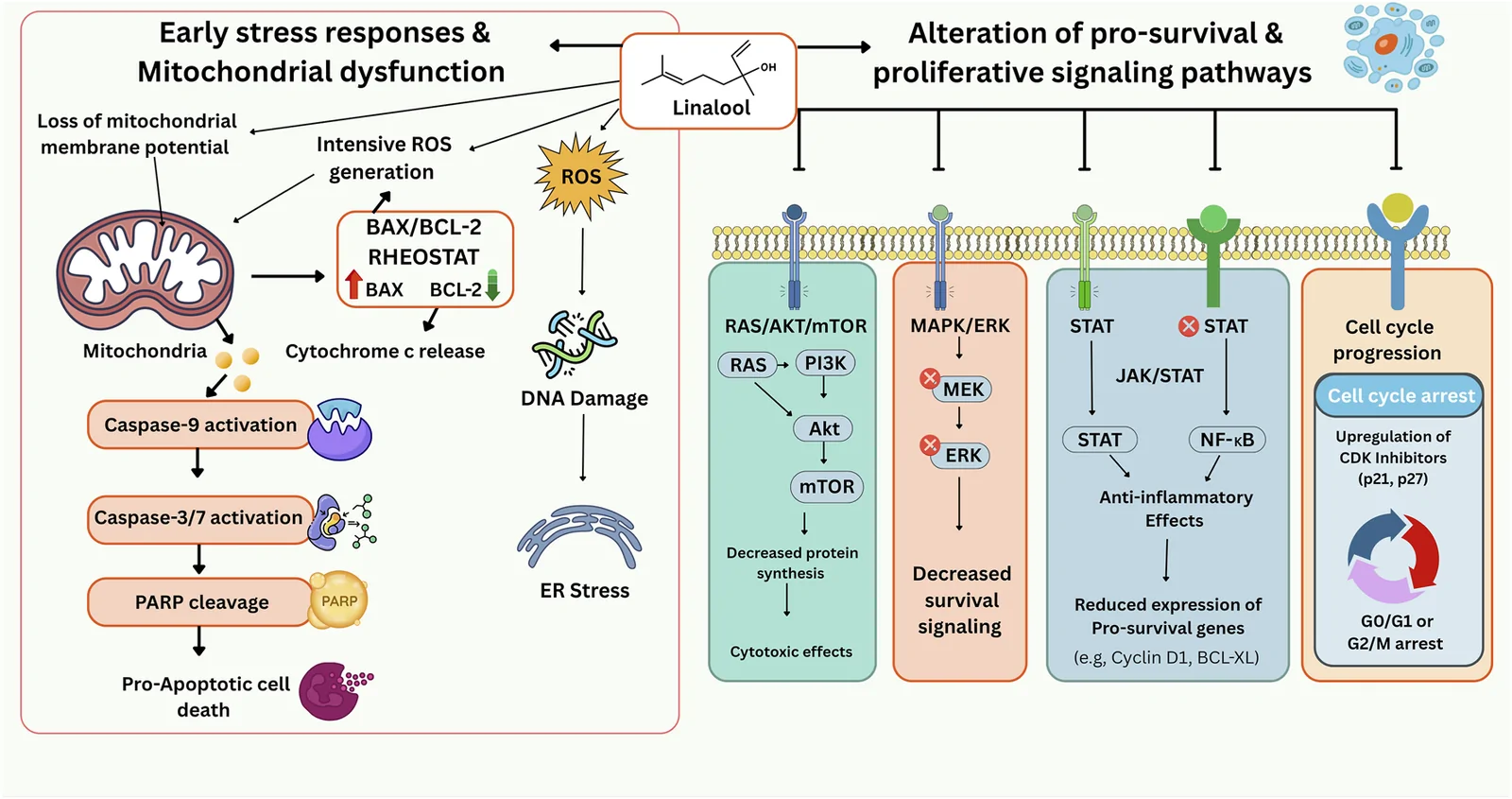
Schematic representation of anticancer mechanisms that occur when linalool triggers mitochondrial dysfunction, oxidative stress, and modulation of survival pathways. Linalool causes loss of mitochondrial membrane potential, generation of reactive oxygen species (ROS), release of cytochrome c, imbalance of anti-apoptotic BCL-2/BAX proteins, activation of caspases, cleavage of poly (ADP-ribose) polymerase (PARP) and apoptosis. It also inhibits the RAS/phosphoinositide 3-kinase/protein kinase B/mammalian target of rapamycin (RAS/PI3K/AKT/mTOR); mitogen-activated protein kinase/extracellular signal-regulated kinase (MAPK/ERK) and Janus kinase/signal transducer and activator of transcription (JAK/STAT) pathways, resulting in decreased survival signaling, decreased pro-survival gene expression, anti-inflammatory properties, and cell-cycle arrest. The mechanisms may differ depending on the type of cancer, experimental model, and conditions of exposure to linalool.

Despite the poor prognosis of epithelial ovarian cancer, more than 70% of patients initially respond to surgery and chemotherapy. However, drug-resistant cancer cells complicate advanced and recurrent disease, creating a need for better-supported therapeutic approaches (Siegel et al., 2012; Landen et al., 2008). In preclinical systems, linalool has shown cytotoxic effects and has been reported to increase doxorubicin uptake in cancer cells, thereby enhancing doxorubicin-associated cytotoxicity (Chang et al., 2014; Miyashita and Sadzuka, 2013; Rodenak Kladniew et al., 2014). In ovarian cancer cell lines (HeyA8, SKOV3ip1, and A2780), linalool nanoparticles were associated with mitochondrial and cytoplasmic redox interactions, intracellular reactive oxygen species production, and caspase-9/caspase-3 activation (Hosseini et al., 2023). In ovarian cancer models, linalool-containing nanoparticles, rather than free linalool alone, were associated with mitochondrial redox changes, intracellular ROS production, and caspase-9/caspase-3 activation (Han et al., 2016). Because these studies used carrier-based systems, the observed effects should be interpreted as formulation-associated effects unless a direct comparison with free linalool and empty carriers demonstrates that the biological response is attributable specifically to linalool (Jabir et al., 2020). In ovarian cancer models, biological effects have been reported for both free linalool and linalool-containing nanoparticle systems. However, findings obtained from essential oils, extracts, or nanoformulations cannot be directly attributed to free linalool unless chemical composition, linalool content, and appropriate carrier controls are reported. Therefore, nanoparticle-associated effects should be interpreted as formulation-dependent responses rather than direct evidence of isolated linalool activity. The proposed molecular mechanism underlying linalool-induced anticancer effects through mitochondrial dysfunction, oxidative stress, and modulation of pro-survival signaling pathways is shown in Figure 2.

Ovarian cancer studies suggest that linalool-containing delivery systems may enhance anticancer responses through oxidative stress, mitochondrial dysfunction, and caspase-mediated apoptosis. A major strength is the evaluation of nanoparticle formulations and combination strategies with chemotherapy, addressing limitations associated with free linalool exposure (Han et al., 2016; Wi et al., 2020). However, attribution of effects specifically to linalool remains challenging because many studies evaluate engineered formulations without adequate free-compound or carrier controls (Han et al., 2016; Jabir et al., 2020). Limited animal validation and exposure data further restrict therapeutic interpretation. Future studies should establish formulation safety, biodistribution, and achievable concentrations to evaluate linalool-based systems as potential sensitizing agents (Shi et al., 2016; Wang et al., 2023).

### 4.3 Lung cancer

Lung cancer is responsible for a high proportion of cancer-related deaths globally, with non-small cell lung cancer accounting for approximately 85% of cases (Bray et al., 2018; Zappa and Mousa, 2016). Smoking remains the primary risk factor and contributes to mutations and signaling changes involving genes such as MYC, Bcl-2, and p53, which are implicated in NSCLC development (Kenzerki et al., 2023). Singh and Meena used *in silico* modeling to predict possible binding of linalool with lung cancer-related markers, including BRAF, and performed computational ADMET analyses (Singh and Meena, 2022). These data should be interpreted as exploratory hypotheses. Experimentally, A549 human lung adenocarcinoma cells were exposed to linalool for 24, 48, and 72 h, and MTT assays were used to estimate cytotoxic effects and IC30 values. The combined computational and experimental approach provides preclinical information, but docking does not independently prove a mechanism of action.

In cigarette smoke–induced lung inflammation models, linalool administration reduced inflammatory-cell infiltration and lowered TNF-α, IL-6, IL-1β, IL-8, MCP-1, and NF-κB-related responses. Linalool also reduced morphological alterations and lung myeloperoxidase activity induced by cigarette smoke and inhibited dose-dependent NF-κB activation (Ma et al., 2015). In both *in vivo* and A549 lung-cell models, linalool increased nuclear Nrf-2 translocation, reduced lung-tissue levels of pro-inflammatory cytokines such as TNF-α and IL-6, and increased expression of Nrf-2–mediated antioxidant enzymes (Wu et al., 2014). Additional molecular studies reported downregulation of pro-inflammatory and metastasis-related genes, including IL-1α, TNF-α, IL-6, COX-2, and vimentin (Nazir et al., 2026). These findings are best described as anti-inflammatory and cytotoxic preclinical endpoints rather than established anticancer efficacy.

In A549 cells, linalool and 1,8-cineole were evaluated using MTT cytotoxicity assays, flow cytometry for apoptosis and cell-cycle analysis, and Western blotting. ROS production and mitochondrial function were examined, with N-acetylcysteine used to explore ROS dependence. Linalool at 2.0 mM inhibited A549 cell migration and was associated with caspase-3 and caspase-9 activation, PARP cleavage, and DNA fragmentation. These results support ROS-dependent mitochondrial dysfunction and apoptosis-associated cytotoxicity *in vitro* (Rodenak-Kladniew et al., 2020). Lung cancer models indicate that linalool influences inflammatory pathways, oxidative balance, mitochondrial function, and apoptosis-related mechanisms. Strengths include the integration of computational predictions, cellular validation, and inflammation-related animal models (Singh and Meena, 2022; Ma et al., 2015; Rodenak-Kladniew et al., 2020). However, computational findings remain hypothesis-generating, and some anticancer effects require millimolar concentrations that may not be achievable systemically (Singh and Meena, 2022; Wang et al., 2023). Anti-inflammatory responses in smoke-induced lung injury models should not be considered direct evidence of anticancer efficacy (Ma et al., 2015; Wu et al., 2014). Translational studies should determine whether targeted delivery approaches can achieve effective lung exposure with acceptable safety profiles (Pereira et al., 2018a; Shi et al., 2016).

### 4.4 Hepatocellular carcinoma

**FIGURE 3:**
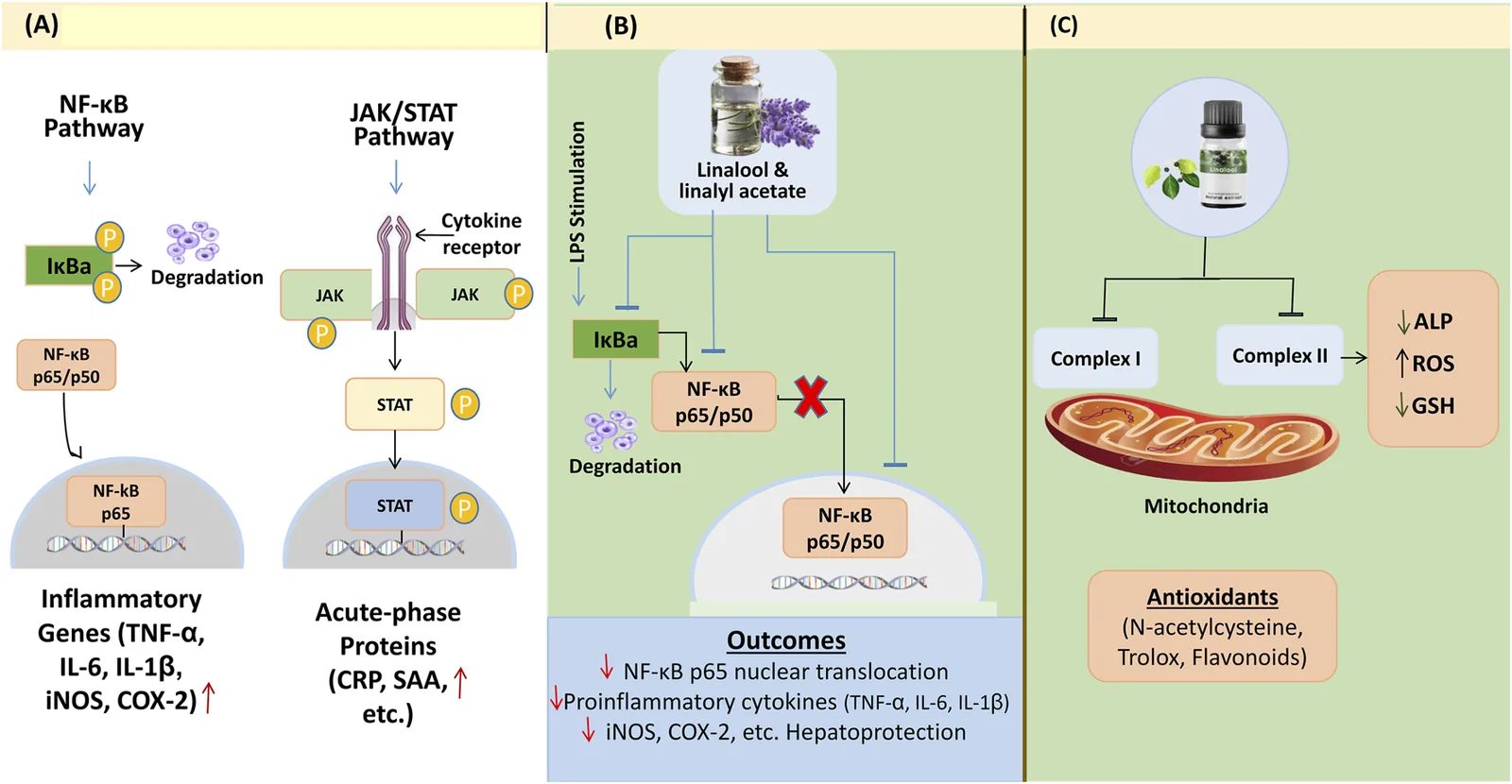
Hepatoprotective and anti-inflammatory mechanisms of linalool. **(A)** Activation of the NF-κB and JAK/STAT pathways induces the expression of inflammatory genes, such as tumour necrosis factor-α (TNF-α), interleukin-6 (IL-6), interleukin-1β (IL-1β), inducible nitric oxide synthase (iNOS) and cyclooxygenase-2 (COX-2), along with the production of acute-phase proteins. **(B)** Linalool and linalyl acetate suppress the NF-κB signaling pathway by blocking IκBα degradation and decreasing the nuclear translocation of NF-κB p65/p50, thereby reducing the expression of pro-inflammatory cytokines and producing hepatoprotective effects. **(C)** Linalool has beneficial effects on antioxidant defenses and mitochondrial function via modulation of mitochondrial complexes I and II, reduction of the generation of reactive oxygen species (ROS) and improvement of glutathione (GSH) status.

Hepatocellular carcinoma (HCC) ranks among the most common cancers globally and is associated with liver cirrhosis, fatty liver disease, hepatitis, alcohol exposure, chemical and toxin exposure, and genetic factors (Huang et al., 2021). Liver inflammation can contribute to cirrhosis and tumor development when hepatocytes and immune cells release pro-inflammatory cytokines such as TNF-α and IL-6. These cytokines activate signaling pathways, particularly NF-κB and JAK/STAT, which further increase inflammatory mediators and acute-phase proteins (Gong et al., 2023). The reported hepatoprotective and anti-inflammatory mechanisms of linalool are presented in Figure 3.

Linalool and linalyl acetate have been reported to attenuate canonical NF-κB signaling in HepG2 inflammatory models. Both metabolites suppressed IκBα phosphorylation and degradation after lipopolysaccharide stimulation, thereby reducing NF-κB p65 nuclear translocation. Western blotting, confocal microscopy, and qPCR analyses supported reductions in NF-κB-mediated inflammatory responses, suggesting anti-inflammatory and hepatoprotective activity *in vitro* (Wu et al., 2025). Linalool also inhibited mitochondrial complex I and complex II activities in HepG2 cells, decreased ATP levels, increased ROS production, and reduced glutathione levels; antioxidants such as N-acetylcysteine, Trolox, and flavonoids reversed some of these effects (Usta et al., 2009). Additional work reported G_0_/G_1_ cell-cycle arrest, reduced cyclin A and Cdk4 expression, increased p21 levels, mitochondrial dysfunction, caspase-3 activation, PARP cleavage, Ras/AKT/mTOR inhibition, and MAPK pathway modulation. These results support cytotoxic and pro-apoptotic mechanisms *in vitro*, but their clinical relevance depends on achievable exposure and selectivity (Rodenak-Kladniew et al., 2018). In hepatocellular carcinoma models, linalool affects mitochondrial function, oxidative stress, cell-cycle regulation, apoptosis, and Ras/MAPK and Akt/mTOR signaling pathways. The strength of current evidence lies in the identification of interconnected molecular responses through biochemical and cellular analyses (Usta et al., 2009; Rodenak-Kladniew et al., 2018). Nevertheless, findings require cautious interpretation because cell-based models may not represent tumor complexity, and effective concentrations may not correspond to achievable hepatic exposure (Rodenak-Kladniew et al., 2018; Wang et al., 2023). Distinguishing selective anticancer activity from general hepatocellular toxicity remains essential. Future studies should evaluate tumor selectivity, hepatic distribution, and exposure-response relationships to clarify the translational potential of linalool (Pereira et al., 2018a; Wang et al., 2023).

### 4.5 Leukemia

Leukemia, with 474,519 cases reported in 2020, ranks as the 12th most common cancer worldwide (Huang et al., 2022). Acute myeloid leukemia is characterized by overproduction of immature myeloblasts affecting blood and bone marrow (Wachter and Pikman, 2024). In T-ALL and B-lymphocyte cell lines (Jurkat, H9, Molt-4, Raji), linalool suppressed proliferation in a dose-dependent manner, with reported IC_50_ values of roughly 20–30 μM, and induced apoptosis-associated and cell-cycle changes. Linalool also arrested U937 leukemia cells in the G_0_/G_1_ phase (Gong et al., 2020). These effects were associated with MAPK modulation, including reduced ERK1/2 phosphorylation and increased JNK activity. Increased CDK inhibitors (p21, p27, p16, p18) and reduced CDKs and cyclins (cyclin D, E, A) were linked to apoptosis-associated increases in the sub-G1 population (Chang et al., 2015). Linalool has also been reported to protect human lymphocytes from benzene-induced cytotoxicity by reducing oxidative stress and preserving organelle integrity, including effects on lipid peroxidation, ROS formation, mitochondrial membrane potential, lysosomal stability, and glutathione redox balance (Gu et al., 2010; Salimi et al., 2022). Leukemia models provide relatively stronger evidence for linalool activity, demonstrating apoptosis induction, cell-cycle regulation, MAPK modulation, and selective effects against malignant cells compared with normal lymphocytes. The inclusion of non-cancer cell comparisons and lower active concentrations in some studies strengthens the biological relevance of these findings (Gong et al., 2020; Gu et al., 2010). However, the evidence remains limited to cell-line models, with insufficient investigation of patient-derived systems, resistance mechanisms, and *in vivo* validation. Translational development should therefore focus on confirming safety, achievable exposure, and potential roles for linalool as a sensitizing or supportive agent in leukemia treatment (Gong et al., 2020; Wang et al., 2023).

### 4.6 Cervical cancer

Cervical cancer remains a major cause of cancer morbidity and mortality among women worldwide (Wu et al., 2022; Arbyn et al., 2020). It is generally classified as adenocarcinoma or squamous cell carcinoma according to the tumor origin (Zivarpour et al., 2021). In HeLa cervical cancer cells, linalool produced cytotoxic and pro-apoptotic effects by inducing G_0_/G_1_ cell-cycle arrest and apoptosis-associated markers. It upregulated cyclin-dependent kinase inhibitors, including p21 and p27, and reduced CDK2 and CDK4 activity. Caspase-3 activation and DNA fragmentation were also reported, supporting an intrinsic apoptosis-related mechanism *in vitro* (Chang et al., 2015). Linalool induces cell-cycle arrest and apoptosis in cervical cancer models through the modulation of cyclin-dependent kinase inhibitors, p53 signaling, and mitochondrial apoptotic pathways. A key strength is the consistent identification of apoptosis-related molecular changes beyond simple viability reduction (Chang et al., 2015). However, evidence is restricted by single-cell-line studies, limited selectivity assessment, and the absence of robust *in vivo* confirmation. Furthermore, unresolved pharmacokinetic limitations prevent the translation of these findings into clinical efficacy (Chang et al., 2015; Wang et al., 2023). Future research should evaluate more representative cervical cancer models, including HPV-associated systems, while incorporating delivery strategies that improve bioavailability and tumor exposure (Pereira et al., 2018a; Shi et al., 2016).

## 5 Preclinical evidence synthesis

The currently available cancer-model literature on linalool consists mainly of *in vitro* studies and a smaller number of animal studies. No eligible human clinical trial was identified that establishes linalool as an anticancer therapy. Therefore, the evidence is described here as preclinical cytotoxic, anti-proliferative, anti-metastatic, or pro-apoptotic activity rather than clinical anticancer efficacy. Linalool and linalool-containing nanoparticles have shown IC_50_ values of 33.14 μg/mL and 22.12 μg/mL, respectively, in the SF-767 glioblastoma cell line, together with reduced PD-L1 expression and increased PTEN expression (Manzoor et al., 2025). Linalool also induced apoptosis and inhibited growth in 22Rv1 prostate cancer cells in both *in vitro* and xenograft models. Western blotting suggested involvement of mitochondrial and death-receptor–related pathways, and xenograft tissues showed reduced Ki-67 and PCNA expression (Zhao et al., 2020).

Linalool (0–2.0 mM) and 1,8-cineole (0–8.0 mM) induced G0/G1 and/or G2/M cell-cycle arrest without reducing normal WI-38 lung-cell viability under the reported conditions. These findings indicate selective preclinical activity in the tested model, but the millimolar concentrations required should be considered when evaluating translational plausibility (Rodenak-Kladniew et al., 2020). In a colon-cancer model, oral linalool at 100 or 200 μg/kg was reported to induce cancer-selective oxidative stress and delayed lipid peroxidation, suggesting a possible preclinical mechanism requiring further validation (Iwasaki et al., 2016).

Linalool was found to increase ROS production (Kim et al., 2014). When the MCF-7 cell line was treated with linalool at 10 μg/mL, minimal cytotoxicity was observed, while treatment with gold nanoparticles (GNPs) at the same concentration showed some cytotoxic effects. More than 70% of the cells died following treatment with LIN-GNPs and functionalized LIN-GNPs-Cys-Ala-Leu-Asn-Asn (CALNN) particles at a concentration of 10 μg/mL. These substances did not significantly affect the HBL cell line. The results suggest that these nanoparticle-based systems may represent a potential source of anti-proliferative and cytotoxic activity in experimental models (Jabir et al., 2019). Although nanoparticle delivery systems may enhance the cellular uptake, stability, and apparent anticancer activity of linalool, the contribution of the carrier material cannot always be excluded. Gold nanoparticles, lipid carriers, and peptide-functionalized systems may independently influence cellular responses. Therefore, comparative studies including free linalool, unloaded nanoparticles, and loaded formulations are required to establish whether the observed effects are specifically mediated by linalool.

**FIGURE 4:**
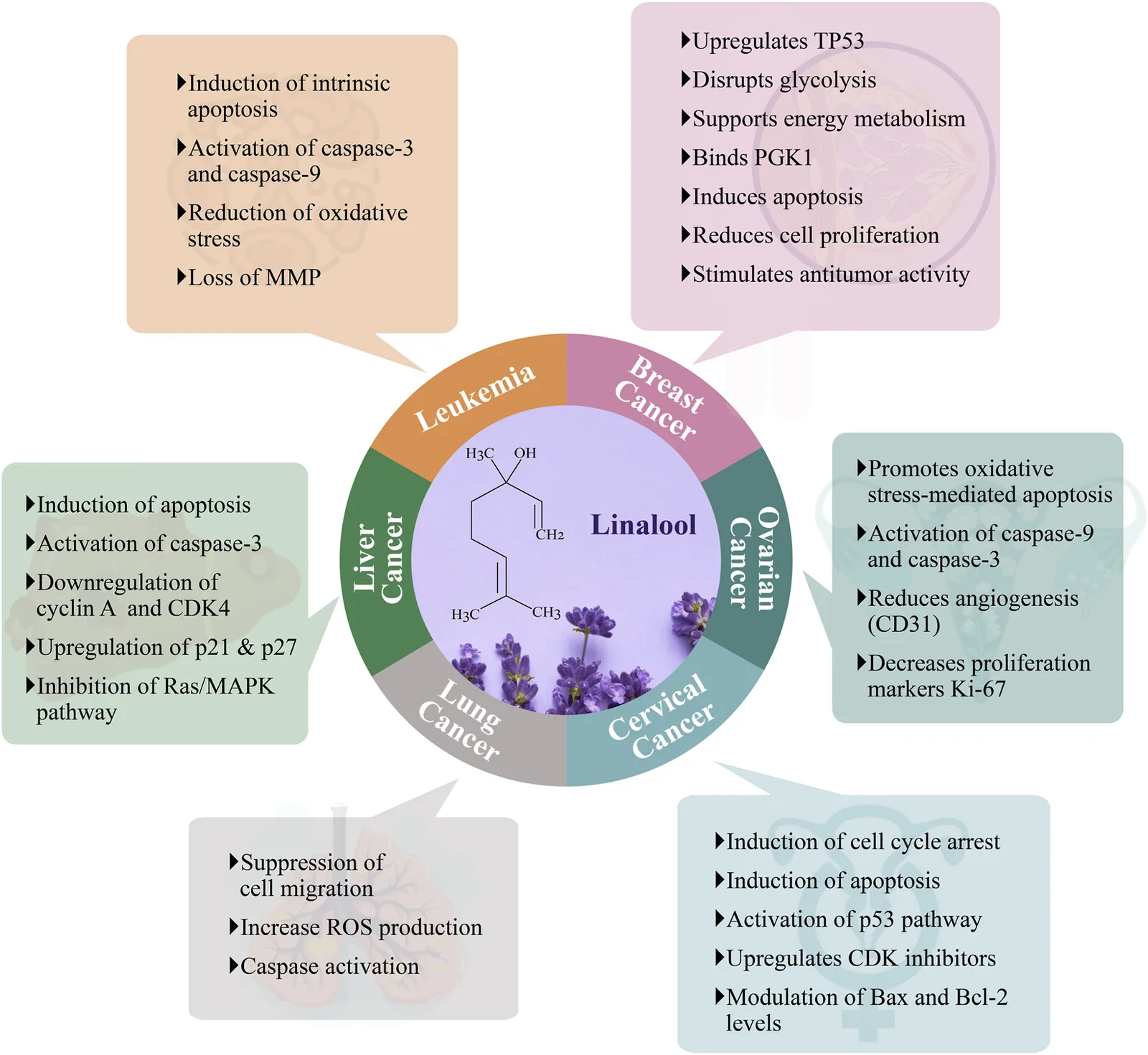
Schematic overview of reported preclinical cytotoxic and pro-apoptotic mechanisms of linalool in different tumor models. The central panel shows the chemical structure of linalool and a representative *Lavandula* spp. source. Surrounding segments summarize reported cellular endpoints in leukemia, breast, ovarian, cervical, lung, and liver cancer models. These mechanisms should be interpreted according to the evidence hierarchy and limitations summarized in Tables 3, 4. In leukemia, linalool induces intrinsic apoptosis through activation of caspase-3 and caspase-9, reduction of oxidative stress, and loss of mitochondrial membrane potential (MMP). In breast cancer, it upregulates TP53, interacts with phosphoglycerate kinase 1 (PGK1), disrupts glycolysis, alters energy metabolism, induces apoptosis, and suppresses proliferation. In ovarian cancer, linalool promotes oxidative stress–mediated apoptosis, activates caspase-3 and caspase-9, reduces angiogenesis (CD31), and decreases the proliferation marker Ki-67. In cervical cancer, it causes cell-cycle arrest and apoptosis through activation of the p53 pathway, upregulation of CDK inhibitors, and modulation of Bax and Bcl-2. In lung cancer, linalool increases ROS production, activates caspases, and suppresses cell migration. In liver cancer, it induces apoptosis through caspase-3 activation, downregulation of cyclin A and CDK4, upregulation of p21 and p27, and inhibition of the Ras/MAPK pathway. For references, see Table 4.

Human T-ALL cell lines (Jurkat), peripheral blood mononuclear cells (PBMCs) from healthy donors, and H9, Molt-4, and B-lymphocyte-derived Raji cells were treated with varying concentrations of linalool (3.75, 7.50, 15, 30, 60, and 120 µM). Cell viability was assessed using a CCK-8 assay, which showed that linalool suppressed T-ALL cell proliferation in a dose-dependent manner. However, linalool did not significantly affect normal PBMCs. It was reported that linalool induces T-ALL cell death through modulation of the MAPK signaling pathway. Factors such as JNK activation and ERK inhibition may contribute to the induction of apoptosis in T-ALL cells. These findings suggest potential preclinical relevance of linalool in T-ALL research models (Gong et al., 2020). The *in vitro* and *in vivo* cancer-related effects of linalool are summarized in Table 4; Figure 4.

**TABLE 4:** Preclinical cancer cell and animal model evidence for linalool: Concentration, model, endpoint, and evidence caveats.

Cancer/model	Cell line or model^1^	Concentration/dose	Reported endpoint(s) with evidence caveat	References
Glioblastoma	SF-767	33.14 and 22.12 μg/mL	Reduced PD-L1 and increased PTEN expression, with cytotoxicity observed in SF-767 cells. Verification of formulation identity, controls, and selectivity is required	Manzoor et al. (2025)
Prostate cancer	22Rv1	2.5 mM	Apoptosis-associated endpoints and reduced Ki-67/PCNA expression in a xenograft context; evidence remains preclinical	Zhao et al. (2020)
Lung cancer	A549	0–2.0 mM	PARP cleavage, DNA fragmentation, G_0_/G_1_ arrest, and increased ROS production; millimolar concentrations should be interpreted cautiously	Rodenak-Kladniew et al. (2020)
Colon cancer	HCT-116	100 or 200 μg/kg	Oxidative stress- and apoptosis-associated endpoints; route, dose, and control conditions require further evaluation	Iwasaki et al. (2016)
Ovarian cancer	HeyA8, A2780par, SKOV3ip1	300 or 600 mg/kg, as reported	Caspase-8/9 activation and ROS-associated endpoints; nanoparticle formulations should be clearly distinguished from effects of free linalool	Kim et al. (2014)
Breast cancer	MCF-7	10 μg/mL LIN-GNPs 10 μg/mL LIN-GNPs-CALNN 10 μg/mL	Cell growth arrest and apoptosis-associated cytotoxicity; effects of gold nanoparticles and peptide functionalization should not be attributed solely to free linalool	Jabir et al. (2019)
Leukemia	Jurkat, H9, Molt-4, Raji, PBMCs, U937	3.75 µM; 7.50 µM; 15 μM; 30 μM; 60 and 120 μM; 2.59 µM	Reduced p-ERK1/2, increased p-JNK, and G0/G1 arrest; *in vitro* evidence with PBMC selectivity reported where available	Gong et al. (2020)
Cervical cancer	HeLa	11.02 μM	G_2_/M arrest with increased p53, p21, p27, p16, and p18 expression; *in vitro* cytotoxic and pro-apoptotic evidence	Chang et al. (2015)
Oral cancer	OECM-1	10 µM	Cell-cycle arrest, loss of mitochondrial membrane potential (MMP), and PI3K/AKT pathway suppression; evidence remains preclinical	Pan and Zhang (2019)
Hepatocellular carcinoma	HepG2	2 μM	Inhibition of mitochondrial complexes I/II, increased ROS production, and reduced ATP and GSH levels; *in vitro* cytotoxicity evidence	Usta et al. (2009)
Skin cancer (UV-related skin-cell model)	HDFa	30 μM	MAPK and NF-κB pathway modulation in a UVB-related skin-cell model; findings indicate photoprotective and anti-inflammatory activity rather than direct anticancer efficacy	Gunaseelan et al. (2016)

1 Cell lines are: 22Rv1, human prostate carcinoma cell line; A2780par, human ovarian carcinoma cell line parental; A549, human lung adenocarcinoma cell line; H9, human T-cell line; HCT-116, human colorectal carcinoma cell line; HDFa, human dermal fibroblasts, adult; HeLa, human cervical carcinoma cell line; HepG2, human hepatocellular carcinoma cell line; HeyA8, human ovarian cancer cell line; Jurkat, human T lymphocyte leukemia cell line; MCF-7, human breast adenocarcinoma cell line; Molt-4, human acute lymphoblastic leukemia cell line; OECM-1, human oral squamous cell carcinoma cell line; PBMCs, peripheral blood mononuclear cells; Raji, human Burkitt’s lymphoma B-cell line; SF-767, human glioblastoma cell line; SKOV3ip1, human ovarian carcinoma cell line, intraperitoneal variant 1; U937, human histiocytic lymphoma/monocytic leukemia cell line. Abbreviations used are: ATP, adenosine triphosphate; CALNN, pentapeptide Cys-Ala-Leu-Asn-Asn; ERK, extracellular signal-regulated kinase; G_0_/G_1_ phase, resting (G_0_) and first gap (G_1_) phases of the cell cycle; GNPs, gold nanoparticles; GSH, glutathione; JNK, c-Jun N-terminal kinase; Ki-67, proliferation marker protein Ki-67; MAPK, mitogen-activated protein kinase; LIN-GNPs, linalool gold nanoparticles; MMP, mitochondrial membrane potential; NF-κB, nuclear factor κ-light-chain-enhancer of activated B cells; PARP, poly (ADP-ribose) polymerase; PCNA, proliferating cell nuclear antigen; PD-L1, programmed death-ligand 1; p-ERK1/2, phosphorylated extracellular signal-regulated kinase 1/2; PI3K, phosphoinositide 3-kinase; p-JNK, phosphorylated c-Jun N-terminal kinase; PTEN, phosphatase and tensin homolog; ROS, reactive oxygen species.

Linalool showed cytotoxic activity compared with 5-FU in the reported assay, with IC_50_ values of 2.59 µM in U937 cells and 11.02 µM in HeLa cells. It was associated with G0/G1 cell-cycle arrest in U937 cells and G_2_/M arrest in HeLa cells, together with increased expression of p53, p21, p27, p16, and p18. In OECM-1 oral cancer cells, the reported IC_50_ was 10 μM, leading to suppression of cyclin-dependent kinase activity (Chang et al., 2015). In contrast, the IC_50_ against non-cancer FR-2 cells was 65 µM. Linalool was also reported to induce cell-cycle arrest, loss of mitochondrial membrane potential, and inhibition of the PI3K/AKT pathway (Pan and Zhang, 2019). The exposure comparisons indicate a clear translational gap for several models. For example, the reported human plasma C_max_ of approximately 0.55 µM is about 180–720-fold lower than the 100–400 µM range used in T-47D cells and about 3,600-fold lower than the 2.0 mM concentration used in A549 migration assays. Lower reported IC_50_ values, such as 2.59 µM in U937 cells and 10–11 µM in HeLa or OECM-1 cells, are closer to the pharmacokinetic range but still require confirmation under physiologically relevant exposure conditions, including protein binding, exposure duration, metabolism, and selectively against non-cancer cells (Camargo et al., 2025).

At concentrations of 0.4 µM and 2 µM linalool, the viability of HepG2 cells decreased by approximately 50% and 100%, respectively. This decrease in HepG2 cell viability is likely due to inhibition of mitochondrial complexes I and II, along with reduced ATP levels. Additionally, linalool increased ROS production while decreasing GSH levels (Usta et al., 2009). In another study, treatment with linalool at a concentration of 30 µM prevented the loss of antioxidant enzyme activities in HDFa cells induced by acute UVB irradiation (20 mJ/cm^2^). Linalool achieved this by inhibiting UVB-induced activation of NF-κB/p65 through activation of IκBα. Furthermore, linalool treatment in HDFa cells influenced the expression of TNF-α, IL-6, IL-10, MMP-2, and MMP-9 induced by UVB exposure. These findings suggest that linalool can protect human skin cells from oxidative damage caused by UVB radiation and modulate MAPK and NF-κB signaling in HDFa cells (Gunaseelan et al., 2016).

## 6 Conventional versus advanced extraction techniques of linalool

Advanced extraction methods such as supercritical fluid extraction (SFE) and steam distillation (SD) are widely used for isolating volatile plant metabolites from botanical materials (Masyita et al., 2022). SFE is valued for its lower solvent burden, preservation of heat-sensitive metabolites, and high product purity (Guta et al., 2024; Pereira et al., 2023), although cost and scale-up remain limiting factors (Chen et al., 2015). Steam distillation remains cost-effective and widely applied, despite longer extraction times and potentially lower yields (Cassel et al., 2009; de Souza et al., 2020; Preedy, 2016). Advanced and conventional extraction methods for linalool and related terpenoids are presented in Table 5.

**TABLE 5:** Advanced and conventional extraction methods for linalool and related terpenoid plant metabolites.

Method	Target plant metabolite/extract	Advantages and limitations	References
Supercritical fluid extraction	Diterpenes	High extraction yield, purity, and selectivity, with preservation of heat-sensitive compounds and natural flavors. Uses non-toxic solvents and promotes complete separation between the solvent and the product	Chen et al. (2024)
Steam distillation	Monoterpenes, Sesquiterpenes	Low investment requirements and operational costs. A clean method that does not require air treatment	de Souza et al. (2020), Preedy (2016)
Hydrodistillation	Monoterpenes, Sesquiterpenes	Uses water as a solvent and does not leave toxic residues at the end of extraction. Cost-effective because it does not require organic solvents and operates at lower temperatures	Sohpal (2018), Ashraf et al. (2020), Tongnuanchan and Benjakul (2014)
Soxhlet extraction	Monoterpenes, Sesquiterpenes, Triterpenes	Compounds with low solubility can be extracted. Large quantities can be extracted using a smaller amount of solvent. Suitable for heat-stable compounds and does not require filtration	Nafiu et al. (2017), Hossain et al. (2014)
Ultrasound-assisted extraction	Linalool, Linalool-terpineol, 4-terpineol	A rapid, simple, and environmentally friendly method with shorter extraction time, lower energy consumption, and high yield and purity. The equipment is relatively inexpensive and easy to operate	Moradi et al. (2018), González et al. (2014), Yolmeh et al. (2014)
Microwave-assisted extraction	Terpenes	Requires less solvent and is less time-consuming. It is safe and environmentally friendly because it reduces solvent use, energy consumption, and chemical waste	Vieira et al. (2017), Darvishzadeh and Orsat (2022)
Solvent-free microwave extraction	Linalool, 4-terpineol	An innovative, clean, and environmentally friendly technique with rapid processing, high yield, lower energy requirements, and high purity	Tigrine-Kordjani et al. (2006), Lucchesi et al. (2004)
Solvent extraction	Monoterpenoids, Linalool, α-terpinene and γ-terpinene, Sesquiterpenes	Provides good recovery of oil and lipid compounds	Mamidipally and Liu (2004)
Subcritical water extraction	Monoterpenes linalool	Reduces extraction time and increases essential oil yield, producing higher-quality products in a shorter time. It is safer and less expensive	Song and Ko (2022)
Enzyme assisted extraction	Monoterpenes, Sesquiterpenes	Improves the bioactive content of essential oils and extracts. Enzyme pre-treatment enhances the efficiency of compound release from plant tissues	Balasubramaniam et al. (2019), Charoensiddhi and Anprung (2010)

Hydrodistillation (HD) serves as an alternative technique, often yielding high-quality extracts with minimal residue, although some chemical degradation can occur (Ashraf et al., 2020; Sohpal, 2018). It allows the extraction of compounds from delicate flowers that may not withstand the high temperatures of conventional distillation above 100 °C. HD is also suitable for extracting compounds that are poorly soluble in water and have low volatility (Tongnuanchan and Benjakul, 2014). Pizani and colleagues improved the extraction rate and purity of linalool by optimizing extraction conditions (Pizani et al., 2022). Similarly, other studies reported that supercritical fluid extraction (SFE) using supercritical CO2 (SC-CO2) enhanced extraction efficiency, providing high yields and purity of linalool (Tang et al., 2021). Various monoterpenes were identified in SC-CO2 extracts of *Salvia officinalis* leaves, including β-myrcene, α-pinene, β-pinene, limonene, and linalool (Jokic et al., 2018).

The Soxhlet extraction technique is widely used for extracting bioactive compounds from plant materials, especially semi-soluble or poorly soluble compounds. It offers high extraction efficiency and relatively low solvent consumption, making it suitable for heat-stable materials (Nafiu et al., 2017; Hossain et al., 2014). However, it is not appropriate for thermolabile compounds and is energy-intensive, with long extraction times and reduced sample throughput (Ridgway, 2012; Mussatto, 2015). Soxhlet extraction using methylene chloride has been reported to extract essential oil (14.45%) from coriander seeds (*Coriandrum sativum*) (Pavlić et al., 2015). In contrast, ultrasound-assisted extraction (UAE) and microwave-assisted extraction (MAE) are more efficient techniques with shorter processing times, higher yields, and lower energy consumption (Rodríguez and Raghavan, 2021). UAE is particularly useful for extracting polyphenols, while MAE has been reported to produce high yields of cannabis oil (16.6–24.6 g per 100 g dry matter) with minimal solvent usage (Addo et al., 2022).

Newer techniques such as solvent-free microwave extraction (SFME) and subcritical water extraction (SWE) are being explored for their environmental friendliness and high productivity (Orsat and Routray, 2018). SFME, which utilizes microwave heating and dry distillation, can produce high-purity essential oils in approximately 20 min, with greater efficiency and energy savings compared to other methods (Addo et al., 2022). Similarly, SWE, which employs water at controlled temperatures and pressures, can provide improved yields in shorter extraction times; for example, coriander has yielded up to 0.8% essential oil using SWE, significantly higher than that obtained by HD methods (Saim et al., 2008; Song and Ko, 2022). Furthermore, enzyme-assisted extraction (EAE) enhances the extraction of bioactive compounds by breaking down plant cell walls with enzymes, resulting in higher yields of oxygenated compounds, particularly in essential oils (Balasubramaniam et al., 2019; Landbo and Mayer, 2001). Overall, these advanced techniques are faster, more environmentally friendly, and often provide higher yields with reduced environmental impact compared with traditional extraction methods.

## 7 Limitations

This review has several limitations that also reflect limitations in the primary literature. First, many studies describe linalool as having anticancer effects when the data more accurately demonstrate cytotoxicity, anti-proliferative activity, apoptosis-related signaling, or anti-inflammatory modulation in preclinical models. Second, molecular docking, ADMET prediction, and network pharmacology are frequently presented as mechanistic evidence, although such approaches are only hypothesis-generating unless validated experimentally. Third, several studies use high micromolar or millimolar concentrations, making pharmacokinetic plausibility uncertain because linalool has low oral bioavailability, rapid metabolism, and limited systemic exposure. Fourth, reporting of controls, positive comparators, exposure duration, selectivity against non-cancer cells, linalool purity, supplier, batch number, and formulation composition is inconsistent. Fifth, studies involving essential oils or plant extracts often do not provide full taxonomic validation, quantified linalool content, or sufficient chemical profiling to attribute observed effects specifically to linalool. Additional limitations include insufficient characterization of long-term toxicity, safety margins, and pharmacodynamic relationships. Although some studies have reported low toxicity in selected models, comprehensive safety evaluation, including repeated-dose toxicity, organ-specific effects, drug interaction potential, and exposure–response relationships, remains limited. Furthermore, the absence of validated pharmacodynamic studies prevents the determination of whether experimentally observed molecular changes correspond to meaningful therapeutic effects. Finally, no robust human clinical evidence currently demonstrates anticancer efficacy. Future work should prioritize standardized chemical characterization, dose response study designs, appropriate positive and negative controls, exposure–response analysis, selectivity testing, reproducible animal studies, and, where justified, rigorously designed clinical evaluation.

## 8 Conclusion and future perspectives

In summary, linalool is a plant-derived monoterpene that exhibits broad preclinical activity, influencing apoptosis, oxidative stress, cell-cycle regulation, and key signaling pathways such as MAPK/ERK, PI3K/AKT, Ras/mTOR, JAK/STAT, NF-κB, and Bax/Bcl-2 networks. The evidence includes studies performed in models of breast, ovarian, lung, liver, leukemia, cervical, prostate, colon, and glioblastoma cancers, predominantly *in vitro*, with limited *in vivo* support and computational predictions, the later of which remains non-confirmatory. Consequently, linalool should be considered a preclinical lead or adjunct candidate rather than an established anticancer agent. Key translational limitations include the use of high micromolar to millimolar concentrations, uncertain pharmacokinetic feasibility due to low bioavailability and rapid metabolism, and inconsistent reporting of dose standardization, controls, and chemical characterization. Studies based on essential oils and extracts further complicate attribution due to incomplete linalool quantification and compositional ambiguity, while *in silico* approaches remain hypothesis-generating only. Future research should prioritize standardized chemical characterization and reporting, including purity, dose, and formulation, rigorous *in vitro* validation with appropriate controls and selectivity testing, and mechanistic confirmation through gene-level and pathway inhibition studies. *In vivo* models should establish pharmacokinetic–pharmacodynamic relationships, tumor exposure, toxicity, and reproducibility. Advanced delivery systems, such as nanoencapsulation, and extraction approaches, such as supercritical extraction, should be evaluated for their capacity to achieve biologically relevant concentrations rather than for yield alone. At present, the evidence base does not support the clinical use of linalool as an anticancer treatment. Therefore, despite promising cytotoxic, apoptotic, and signaling-related effects observed in experimental models, current evidence remains exclusively preclinical and does not support the clinical application of linalool as an anticancer therapy. Future investigations should prioritize pharmacokinetic–pharmacodynamic evaluation, standardized formulations, toxicity assessment, and carefully designed clinical studies before therapeutic conclusions can be established. The central translational gap is whether concentrations associated with cytotoxic or pro-apoptotic activity *in vitro* can be achieved safely, selectively, and reproducibly in humans. Ultimately, well-designed early-phase clinical studies are necessary to assess safety, pharmacokinetics, and translational feasibility before any anticancer application can be substantiated.
