# Supplementary material for: Linalool in cancer pharmacology: pharmacokinetic limitations and preclinical cytotoxic evidence for translational development
Source: Front Pharmacol. 2026 Aug 4;17:1891927. doi: 10.3389/fphar.2026.1891927 (PMC13527556; doi:10.3389/fphar.2026.1891927)
Supplement: Supplementary file 1 [file Table1.docx]

**Supplementary Table 1**: Structured quality and risk-of-bias appraisal of included linalool cancer-model studies

| **Study (Citation)^1^** | **Material category** | **Material identity reported** | **Purity reported** | **Dose/concentration** | **Exposure duration** | **Positive control** | **Negative /vehicle control** | **Non-cancer selectivity** | **Formulation details** | **Statistical robustness** | **Overall confidence** |
| --- | --- | --- | --- | --- | --- | --- | --- | --- | --- | --- | --- |
| Hsu et al., 2014 | Pure compound | Linalool | NR | 100–400 µM (IC_50_ ~224 µM) | 24 h | NR | NR | NR | Pure compound | Flow cytometry and viability assays reported | Moderate |
| Elbe et al., 2022 | Pure compound | Linalool | NR | Dose-dependent (cell-line study) | NR | NR | NR | Partial (MCF-7 vs MDA-MB-231 sensitivity) | Pure compound | Microscopy and Annexin V assay | Moderate |
| Gong et al., 2020 | Pure compound | Linalool | NR | 3.75–120 µM (IC_50_ ~20–30 µM in leukemia lines) | NR | NR | NR | Yes (PBMC comparison) | Pure compound | CCK-8 and pathway analysis | High (relative) |
| Usta et al., 2009 | Pure compound | Linalool | NR | 0.4–2.0 µM (HepG2) | NR | NR | NR | NR | Pure compound | Mitochondrial and ROS assays | Moderate |
| Rodenak-Kladniew et al., 2020 | Pure compound | Linalool and cineole | NR | 0–2 mM | 24–72 h | NAC used (ROS control) | Yes (WI-38 cells) | Yes | Pure compound | Western blot and apoptosis assays | High |
| Chang et al., 2015 | Pure compound | Linalool | NR | ~2.59 µM (U937), 11.02 µM (HeLa) | NR | 5-FU comparison | NR | Limited | Pure compound | Cell cycle and apoptosis markers | High |
| Jabir et al., 2019 | Nano-formulation | Linalool-GNPs | NR | 10 µg/mL | NR | NR | NR | Partial (HBL non-cancer line) | Gold nanoparticle conjugates | Cytotoxicity assay | Moderate |
| Zhao et al., 2020 | Pure compound / in vivo | Linalool | NR | NR | NR | NR | NR | Yes (xenograft and normal markers) | Systemic administration | Western blot and xenograft data | High |
| Pan et al., 2019 | Pure compound | Linalool | NR | NR | NR | NR | NR | NR | Pure compound | PI3K/AKT pathway analysis | Moderate |
| Iwasaki et al., 2016 | Pure compound | Linalool | NR | 100–200 µg/kg (oral) | NR | NR | NR | Suggested (oxidative stress selectivity) | In vivo oral administration | Limited mechanistic validation | Low–Moderate |
| Manzoor et al., 2025 | Nano-formulation | Linalool nanoparticles | NR | 33.14 µg/mL | NR | NR | NR | Not fully reported | Nanoparticle system | PD-L1/PTEN expression | Moderate |

^1^ for references see main text, NR, not reported.
